# Molecular mechanisms involved in regulating protein activity and biological function of MST3

**DOI:** 10.1186/s13008-023-00090-x

**Published:** 2023-05-18

**Authors:** Jing Qiu, Junzhi Xiong, Lu Jiang, Xinmin Wang, Kebin Zhang, Hua Yu

**Affiliations:** 1 Department of Pharmacy, Xinqiao Hospital, Army Medical University, Chongqing, China; 2 Clinical Medical Research Center, Xinqiao Hospital, Army Medical University, Chongqing, China

**Keywords:** MST3, Protein activity, Post-translational modification, Biological function, Disease progression

## Abstract

Mammalian sterile 20-like (Ste20-like) protein kinase 3 (MST3) or serine/threonine-protein kinase 24 (STK24) is a serine/threonine protein kinase that belongs to the mammalian STE20-like protein kinase family. MST3 is a pleiotropic protein that plays a critical role in regulating a variety of events, including apoptosis, immune response, metabolism, hypertension, tumor progression, and development of the central nervous system. The MST3-mediated regulation is intricately related to protein activity, post-translational modification, and subcellular location. Here, we review the recent progress on the regulatory mechanisms against MST3 and its-mediated control of disease progression.

## Introduction

Sterile 20 (STE20) is a serine/threonine protein kinase family that was originally discovered in the budding yeast. Presently, 28 mammalian STE20-like (MST) kinases, homologs to yeast STE20, have been identified [1]. According to the relative location of the kinase domains, these are divided into two families, namely, the p21-activated kinase (PAK) family (COOH terminal kinase domain) and germinal center kinase (GCK) family (NH_2_ terminal kinase domain). In mammals, the five characterized MST family kinases can be divided into two subgroups, namely, GCK-II (MST1 and MST2) and GCK-III (MST3, MST4, and YSK1) [2]. It is reported that the MST family numbers are closely related to the regulation of a variety of biological activities, such as cytoskeletal organization, cell motility, apoptosis, and central nervous system (CNS) development (Table 1). At present, increasing attention has been paid to MST3 regarding its roles in modulating apoptosis, immune response, metabolism, hypertension, tumor progression, and CNS development [2–5]. The MST3-mediated regulation of disease progression is closely associated with protein activity, which is affected by protein cleavage, subcellular distribution, and post-transcriptional modification [6–8]. Therefore, in this review, we summarize the recent progress on the regulatory mode against MST3 and the mechanisms underlying the MST3-mediated control of disease development.

**Table 1:** Similar or different biological functions among MST kinases

MST kinases	Biological functions	References
Similarities (MST1-4 and YSK1)	Cytoskeleton organization	[2]
	Cell polarity and migration	[9]
	Apoptosis	[10, 11]
	Proliferation and migration of cancer cells Immune regulation	[12, 13] [14]
	CNS development	[5]
Differences		
MST1/MST4	Autophagy	[12, 15, 16]
MST1/2	Glycogen storage	[17]
MST1/2	Stem cell function	[18]
MST1/YSK1	Pancreatic cell homeostasis, and insulin secretion	[2]
MST1/3/4/5	Lipid metabolism	[2, 9]
MST3	Hypertension	[19, 20]
MST4	Radioresistance	[12]
YSK1	Golgi integrity	[21]

## Homology of MST kinases

**Fig. 1:**
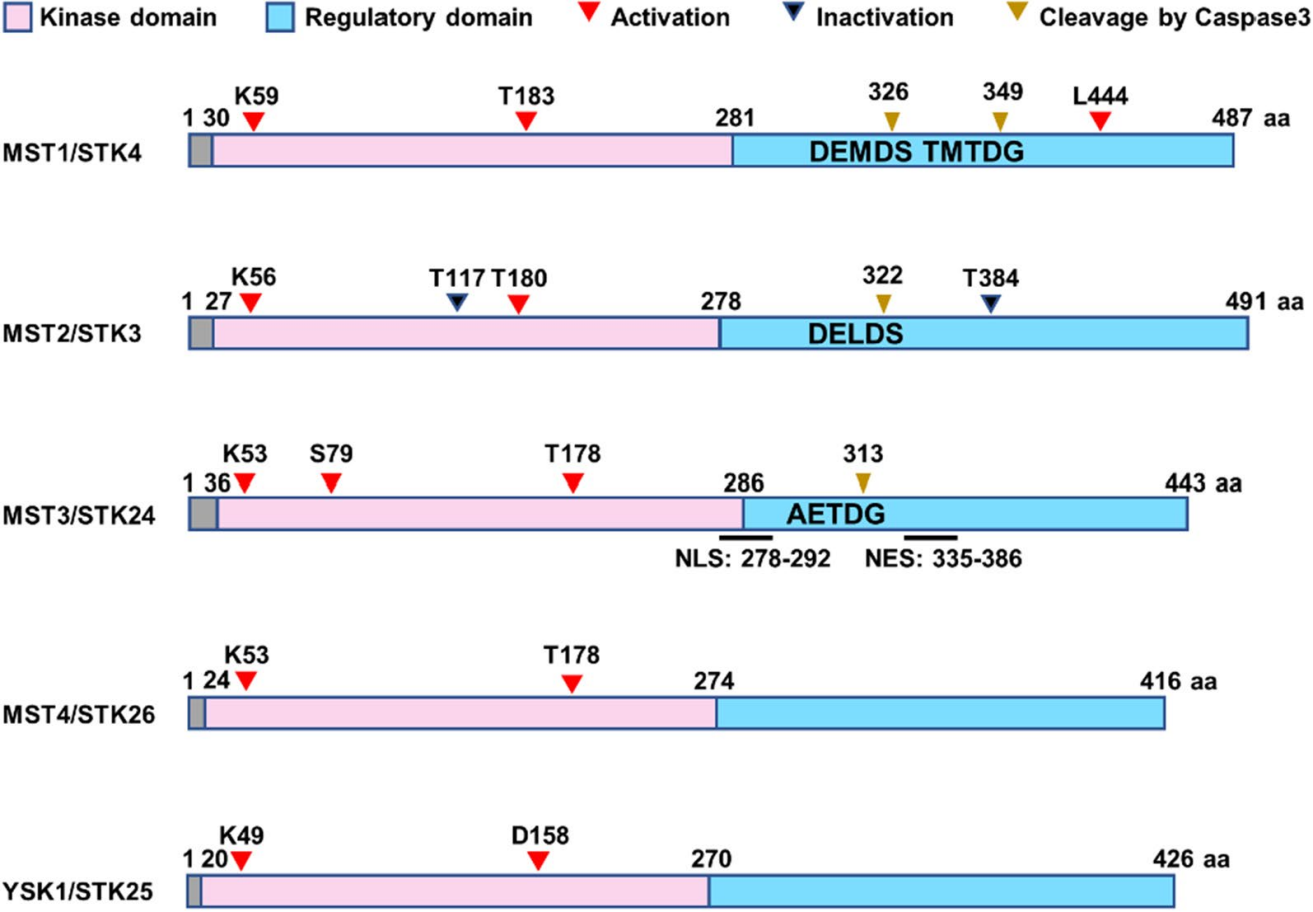
Protein domain and kinase sites of MSTs

**Fig. 2:**
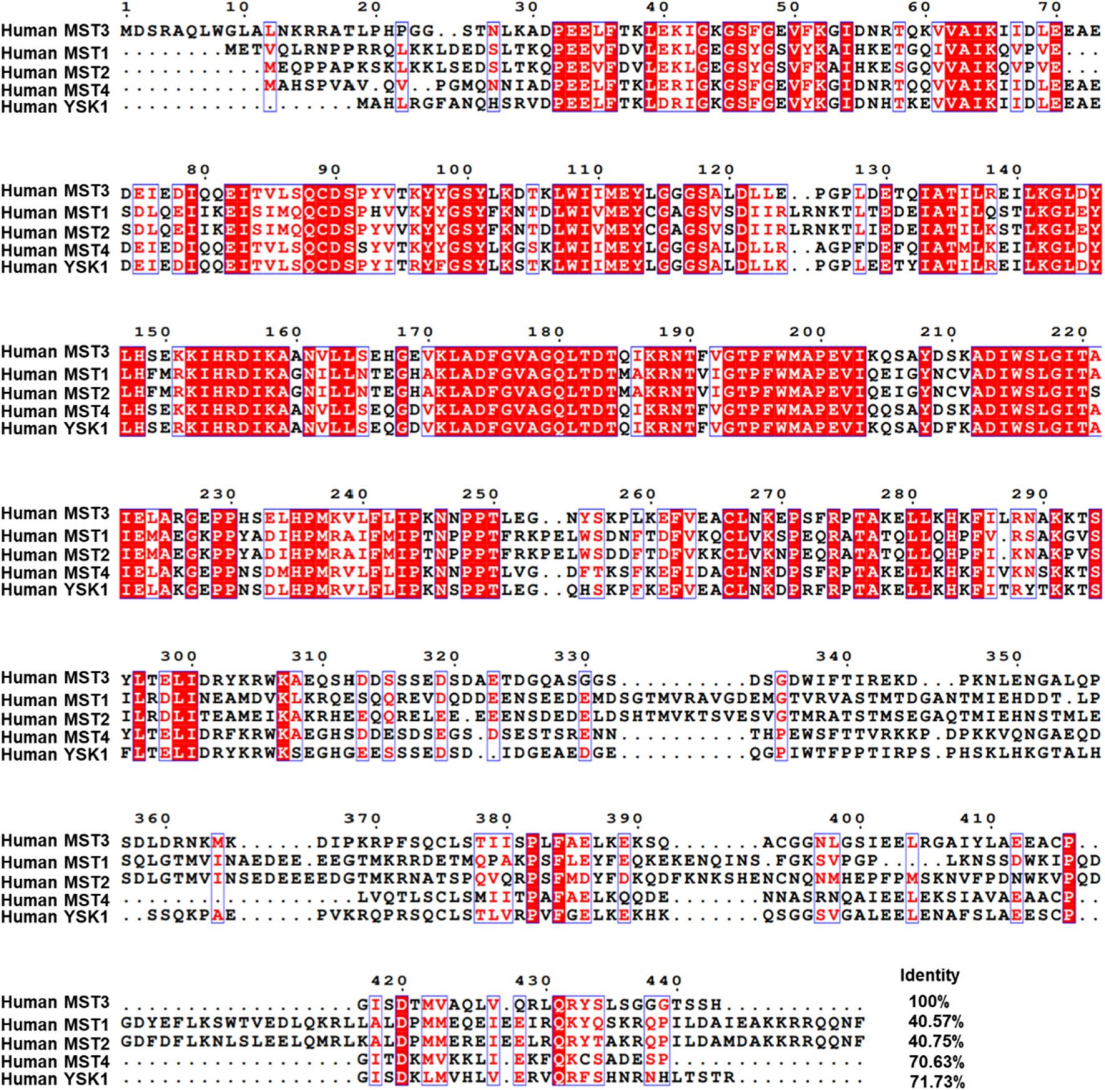
Sequence alignment of human MST family protein. Multiple alignments were carried out using UniProt-Align (https://www.uniprot.org/align). The alignment was drawn using ESPript 3.0 (http://espript.ibcp.fr/ESPript/cgi-bin/ESPript.cgi). MST1 (accession no. UniProtKB-Q13043); MST2 (accession no. Q13188); MST3 (accession no. Q9Y6E0); MST4 (accession no. Q9P289); YSK1 (accession no. O00506)

**Fig. 3:**
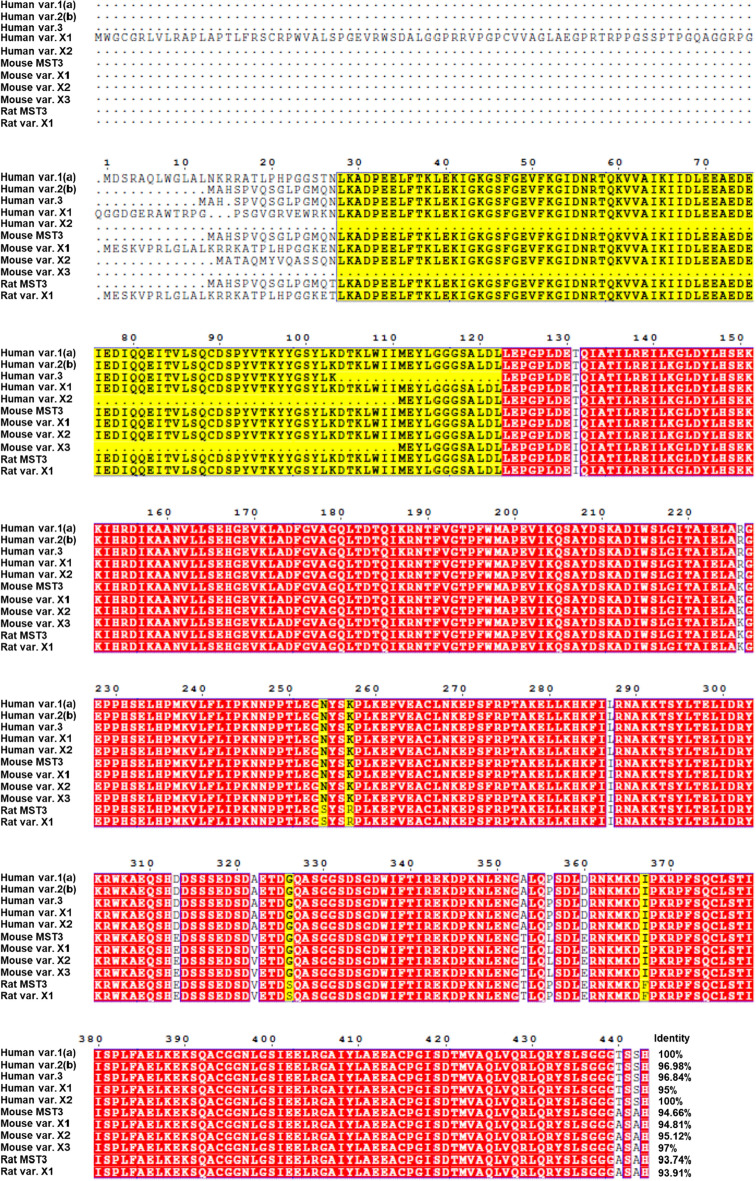
Sequence alignment of MST3 isoforms from human and mouse species. Multiple alignments were carried out using UniProt-Align (https://www.uniprot.org/align). The alignment was drawn using ESPript 3.0 (http://espript.ibcp.fr/ESPript/cgi-bin/ESPript.cgi). Human MST3 variant 1(a) (canonical sequence; accession no. NP_003567.2); MST3 variant 2(b) (accession no. NP_001027467.2); MST3 variant 3 (accession no. NP_001273578.1); MST3 variant X1 (accession no. XP_016876283.1); MST3 variant X2 (accession no. XP_024305194.1); mouse MST3 (canonical sequence; accession no. NM_145465.2); mouse MST3 variant X1 (accession no. XM_017315988.2); mouse MST3 variant X2 (accession no. XM_011245043.4); mouse MST3 variant X3 (accession no. XM_006518918.3); rat MST3 (accession no.NP_001120966.1); rat MST3 variant X1 (accession no. XP_038949467.1)

The MST kinases contain an N-terminal kinase domain and a C-terminal regulatory domain. In human MST3, the N-terminal kinase domain is located at 36–286 amino acids, whereas the C-terminal regulatory domain is located at 287–443 amino acids [22] (Fig. 1). Sequence alignment revealed that human MST3 shares a nearly 70% sequence identity with MST4 and YSK1 while sharing a nearly 40% identity with MST1 and MST2 (Fig. 2). The MST proteins contain high sequence identity at the N-terminal kinase domain but not at the C-terminal domain. Human MST3 has five variants, whereas mouse MST3 has four isoforms. The high rate of the identity of canonical sequences of MST3 among humans, mice and rats is up to 93% (Fig. 3).

## Subcellular distribution

Under normal conditions, MST3 is localized predominantly in the cytoplasm. During apoptosis, the activated caspase-3 cleaves MST3 at the junction of the N- and C-terminal domain (AETD^313^G), following which the truncated MST3 (MST3/N) is translocated into the nucleus [22]. The nuclear localization sequence (NLS) at the C-terminus of its kinase domain (residues 278–292) is required for the intranuclear translocation of MST3 [23], whereas a nuclear export signal (NES) is postulated to be in the C-terminal regulatory domain (amino acids 335–386) (Fig. 1) [23]. It is reported that the myristoylation of MST3 induces the diffusion in the cytosol or translocation into the nucleus via its nuclear localization sequence [24]. These results suggest that the subcellular location of MST3 can be modulated by the diverse cleavage or post-translational modification.

## Kinase activity sites, modifications, and interactions

The MSTs are serine/threonine protein kinases that promote phosphorylation or activation of substrate proteins by transferring phosphate groups from GTP or ATP to the serine or threonine residue of target proteins. The mutants of MST1 K59R, MST2 K56R, MST3/MST4 K53R and YSK1 K49R and D158A display deficient kinase activity [22, 25, 26]. In contrast, the phosphorylation of MST2 Ther117 or Ther384 by Akt renders the kinase inactive [27] (Fig. 1). The activation of MST3 is associated with post-translational modifications, including autophosphorylation, phosphorylation, and myristoylation. Thr178 is the conserved autophosphorylation site of STE20-like kinases [28]. The mutation of threonine to alanine at codon 178 of MST3 or MST4 leads to the deficiency of kinase activity [12, 28]. Although MST3 also autophosphorylates at codon Thr328, the phosphorylation at this residue does not affect the kinase activity of MST3 [29]. In addition, the phosphorylation of MST3 at Lys53 [19, 30] or Ser79 [31] is essential for the kinase activity of MST3 (Fig. 1).

Recently, several upstream kinases and regulators have been reported to regulate the activation of MST3. The cyclin-dependent kinase 5 (Cdk5) that phosphorylates MST3 at Ser79 is essential for the activity of MST3 [31]. A novel isoform of MST3 (MST3b) with a different 5' coding region from MST3 (strictly expressed in the brain), is effectively phosphorylated by cyclic AMP-dependent protein kinase (PKA) at Thr18 (this residue is absent in MST3) [32]. Although MST3 can also be phosphorylated at tyrosine following treatment with a tyrosine phosphatase inhibitor (PV), the tyrosine modification does not alter the activity of MST3 [33]. In addition, MST3 can be myristoylated, which could avoid the binding of its negative regulatory domain with the catalytic domain, resulting in a constitutively active enzyme [24].

Apart from post-translational modification, the activity of MST3 is also modulated by a series of cellular activities, such as caspase-mediated cleavage and interaction with regulators (Table 2). Caspase 3-mediated cleavage of MST3 at the junction of the N-terminal kinase domain and C-terminal regulatory domain activates its intrinsic kinase activity by removing the negative regulatory domain [22]. The binding of MST3 with its master regulator MO25 scaffolding protein stimulates its kinase activity three- to four-fold [34]. In contrast, striatin-interacting phosphatase and kinase (STRIPAK) complex components, protein phosphatase 2A (PP2A) [35] or FAM40A [36], inactivates MST3 by dephosphorylating its activation loop.

**Table 2:** Regulators involved in modulation of MST3 kinase activity

Upstream kinase/regulator	Regulatory mode	Biological function	References
Cdk5	Phosphorylate MST3 at Ser79	Neuronal migration	[31]
PKA	Phosphorylate MST3b at Thr18		[32]
Caspase 3	Cleave MST3 at AETD^313^G	Apoptosis	[22]
MO25	Bind with MST3		[34]
PP2A	Dephosphorylate and inactivate MST3	Cell migration	[35]
FAM40A	Dephosphorylate and inactivate MST3	Cell migration	[14]

## Roles and regulatory mechanisms of MST3 in disease progression

### MST3 and apoptosis

**Fig. 4:**
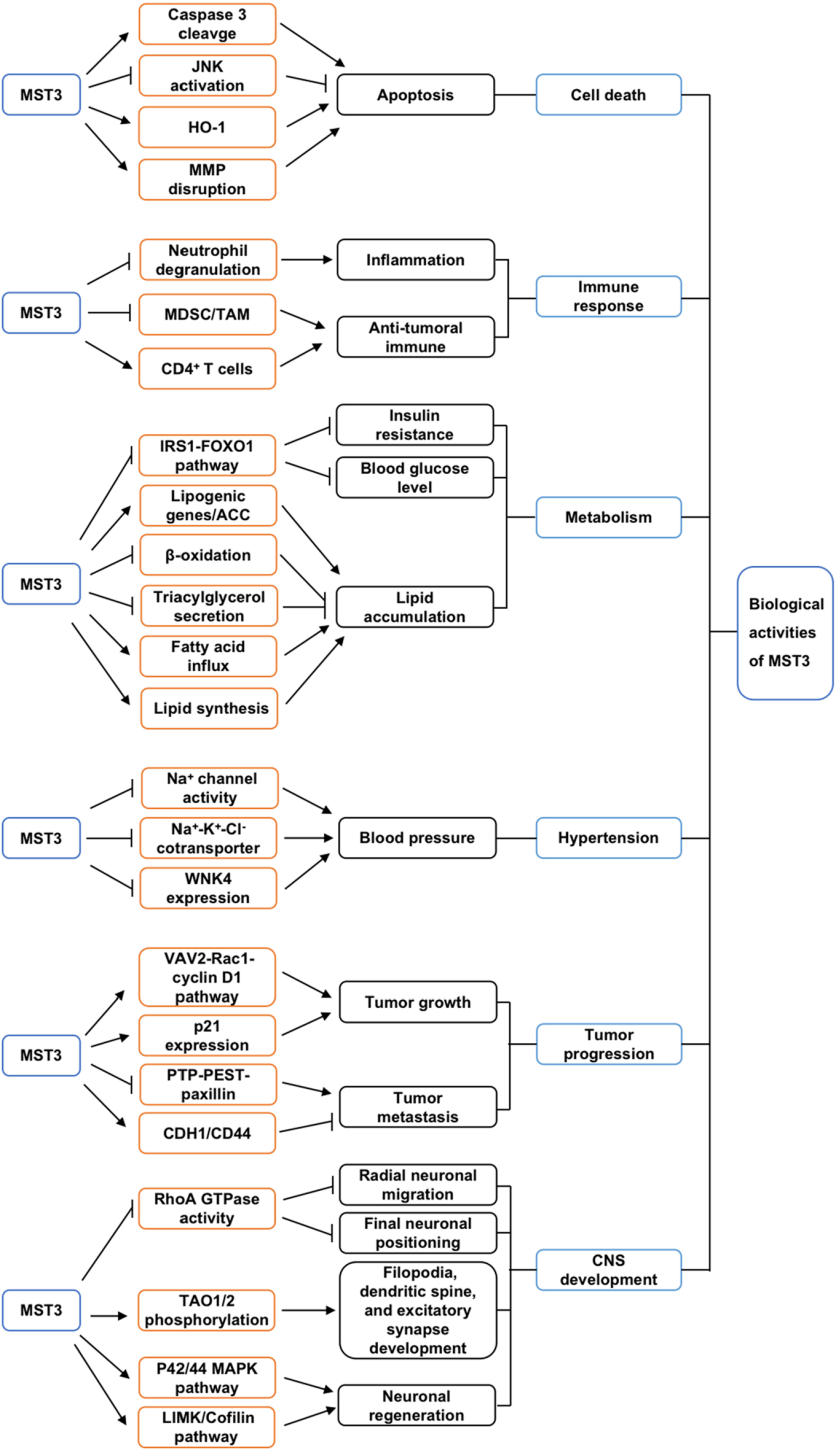
Schematic diagram of MST3-mediated regulation

Apoptosis is a form of programmed cell death that is critical for maintaining cellular physiologic homeostasis. Apoptosis is triggered by a series of effectors (e.g., caspases 3 and 8) and regulators through two major pathways, namely, extrinsic (death receptor-mediated) and intrinsic (mitochondria-mediated) pathways. Researchers have demonstrated that MST3 is closely related to the regulation of apoptosis (Fig. 4 and Table 3). In response to staurosporine, MST3 triggers apoptosis by activating caspase 3 (cleavage of caspase 3), a key player in activating both extrinsic and intrinsic apoptotic pathways by cleaving several downstream cell survival-associated proteins [3]. Moreover, Wu et al. reported that MST3 is overexpressed in placental trophoblasts during labor-induced oxidative stress. The overexpression of *MST3* in the human trophoblast cell line 3A-sub-E promotes caspase 3-mediated apoptosis [30]. In line with this result, researchers reported that MST3 triggers cell death in the hydrogen peroxide (H_2_O_2_)-treated human colon carcinoma HCT116 cell line by suppressing the JNK survival pathway and up-regulating cytoprotective HO-1 (heme oxygenase-1) [6]. Further analysis demonstrated that the MST3 kinase activity is essential for H_2_O_2_-induced apoptosis of cells because the kinase-dead mutant of MST3 (Lys53 to Arg53) displayed an impaired ability to induce apoptosis than the wild-type MST3 [30]. In addition to the caspase-mediated canonical apoptosis, MST3 activates the caspase-independent apoptotic pathway in human cervix HeLa cells by promoting the nuclear translocation of apoptosis-inducing factor and endonuclease G by disrupting the mitochondrial membrane potential (Δψm) [3].

**Table 3:** Biological activities of MST3 and the underlying mechanisms

Biological function	Regulatory mechanism	References
Apoptosis		
Induce apoptosis	Activate caspase 3 in human trophoblast cells	[30]
	Suppress JNK in human colon carcinoma cells	[6]
	Upregulate cytoprotective HO-1 in colon carcinoma cells	[6]
	Disrupt mitochondrial membrane potential in human cervix HeLa cells	[3]
Immune regulation		
Inhibit neutrophil-mediated inflammatory response	Inhibit neutrophil degranulation	[37]
Promote anti-tumoral immune response	Associate with decreased ratio of MDSCs and TAMs in gastric tumor tissue	[38, 39]
	Associate with increased percentage of CD4^+^ T cells in gastric tumor tissue	[38, 39]
Metabolism		
Increase insulin resistance and blood glucose levels	Deactivate IRS1-FOXO1 pathway	[40]
Promote lipid accumulation	Increase the expression of lipogenic genes and ACC	[41]
	Inhibit β-oxidation and triacylglycerol secretion	[42]
	Increase fatty acid influx and lipid synthesis	[42]
Hypertension		
Maintain Na^+^/K^+^ homeostasis and blood pressure stability	Inhibit Na^+^ channel and Na^+^-K^+^-Cl^−^ cotransporter activities	[20, 43]
	Suppress WNK4 expression	[43]
Tumor progression		
Promote breast cancer growth	Activate VAV2-Rac1-cyclin D1 pathway	[4]
Promote gastric cancer growth	Enhance p21 expression	[7]
Inhibit migration of adenocarcinoma	Inhibit paxillin phosphorylation via PTP-PEST	[28]
Suppress migration of gastric cancer	Increase expression of CDH1 (E-Cadherin) and CD44	[38]
CNS development		
Promote radial neuronal migration and final neuronal positioning	Suppress Rho-GTPase activity of RhoA	[31]
Promote the development of filopodia, dendritic spine and excitatory synapse	Phosphorylate TAO1/2 kinases	[44]
Promote neuronal regeneration	Activate P42/44MAPK and LIMK/Cofilin pathway	[45]

Although MST3 has been proved to trigger apoptosis through the caspase effector, it can also be cleaved by caspase 3 at the domain (AETD^313^G) during anti-Fas antibody- or staurosporine-induced apoptosis in Jurkat cells [22]. The cleavage of MST3 by caspase 3 causes nuclear accumulation of the active kinase domain of MST3 and increased apoptosis induced by the truncated MST3 [22]. These results suggest that MST3 and caspase 3 form a feedback loop during the initial stages of apoptosis by modulating the protein cleavage-mediated enhanced activity. However, this speculation requires further investigation.

### MST3 and immune regulation

Reports have revealed that the MST kinase family members, namely, MST1 and MST2, are the important components of the immune-associated Hippo pathway [14]. MST1 and MST2 play crucial roles in regulating both innate and adaptive immune response-related activities, such as T cell homeostasis, lymphocyte trafficking, antiviral immune signaling [46, 47], and CD8α^+^ dendritic cell activation [14]. Neutrophils are the first responders to inflammation and infection; they migrate to inflammatory sites and execute the program of degranulation to release antimicrobial molecules or cytotoxic agents [48]. Zhang et al. reported that the deficiency of STK24 enhances the degranulation of neutrophils. The STK24 binds to UNC13D and suppresses UNC13D-lipid binding and granule docking, thus inhibiting the exocytic process of neutrophil degranulation [37], thereby indicating a critical function of MST3 in promoting host immune response. In addition, the number of immune inhibitory myeloid-derived suppressor cells (MDSCs) and tumor-associated macrophages (TAMs) were increased, whereas the number of tumoricidal CD4^+^ T cells was decreased in the *STK24* knockout gastric tumor sections, indicating that MST3 promotes antitumoral immune response [38, 39]. Nevertheless, the underlying mechanisms need further investigation.

### MST3 and metabolism

Recently, a series of studies have unveiled a previously unknown function of GCKIII kinases in metabolic regulation [2]. As a member of the GCKIII family protein, MST3 has lately been demonstrated to increase insulin resistance and blood glucose levels in mice fed with an obesity-promoting high-fat diet (HFD) [40]. Knockout of *MST3* in these mice led to impaired hyperglycemia, hyperinsulinemia, and insulin resistance. Mechanistic analysis revealed that a lack of MST3 in both cultured liver cells and the livers of animals after HFD activates the insulin signaling pathway downstream of IRS1 by inhibiting forkhead box (FOX)O1-mediated downregulation of genes encoding gluconeogenic enzymes [40]. In addition to the regulation of insulin signaling, studies have reported that MST3, MST4, and STK25 are exclusively localized around intracellular lipid droplets and increase fat accumulation in human hepatocytes as well as the initiation and progression of nonalcoholic fatty liver disease (NAFLD) [9, 41]. Mice treated with antisense oligonucleotides (ASOs) targeting MST3 effectively ameliorated HFD-induced nonalcoholic fatty liver disease (NAFLD)-associated liver steatosis, inflammation, fibrosis, and hepatocellular damage [41]. Mechanistically, MST3 ASOs inhibit the expression of lipogenic genes, as well as acetyl-CoA carboxylase (ACC) protein abundance, leading to reduced lipotoxicity-mediated oxidative and endoplasmic reticulum stress in the liver of obese mice [41]. Similarly, researchers unveiled that MST3 modulates the dynamic metabolic balance of liver lipid catabolism versus lipid anabolism. Knockdown of *MST3* decreased the accumulation of lipids in human hepatocytes by stimulating β-oxidation and triacylglycerol secretion while suppressing fatty acid influx and lipid synthesis [42]. Moreover, recent study reported that all 28 STE20 kinases including MST3 phosphorylate the energy metabolism-related protein kinases, AMP-activated protein kinase (AMPK) and the salt-inducible kinase 3 (SIK3) [49]. The MST3 or the brain expressed MST3b isoform phosphorylates AMPKα1-T183 and SIK3-T221 [50].

### MST3 and hypertension

A combination of defective renal salt and water excretion and increased salt intake frequently contributes to hypertension. Lu et al. disclosed that MST3 is a stress-regulated kinase that maintains sodium homeostasis after a high-salt diet and protects the development of hypertension in mice. The MST3 protein expression is markedly reduced in the kidneys of spontaneously hypertensive rat (SHR) kidneys, whereas this level was elevated when normal mice were administered a high-salt diet [19]. In vitro study unveiled that under hypertonic stress (900 mOsm/L hyperosmolar NaCl medium), the wild type-MST3-MDCK (Madin–Darby Canine Kidney) cells survived. In contrast, the KD-MST3-MDCK (K53R kinase-dead MST3) cells could not resist the hypertonic stress [19], suggesting that MST3-mediated maintenance of sodium homeostasis requires its kinase activity. Further analysis using mice with MST3 hypomorphic mutation demonstrated that the MST3^−/−^ mice exhibit hypernatremia, hypokalemia, and hypertension, and MST3 maintained Na^+^ homeostasis and blood pressure stability by regulating epithelial Na^+^ channel (ENaC) [20]. Moreover, Chan et al. reported that MST3/STK24 is expressed primarily in the medullary thick ascending tubule (TAL) and at lower levels in the late distal convoluted tubules (DCTs) [43]. The hypertension and lower urinary Na^+^ excretion found in MST3^−/−^ mice is associated with increased ENaC activity, WNK4 (with-no-lysine 4) expression, and NKCC2 (Na–K-Cl cotransporter) S130 phosphorylation [43], indicating that MST3 participates in maintaining the Na^+^/K^+^ homeostasis in response to K^+^ loading by inhibiting WNK4 expression, NKCC2, and ENaC activity.

### MST3 and tumor progression

Recently, studies have shown that MST3 deregulation is associated with cancer cell migration and metastasis. *MST3* is overexpressed in the tumor tissues of patients suffering from the human breast [4] and gastric cancer [7]. The overexpression of *MST3* predicts poor prognosis in these cancer patients [4, 7], suggesting that MST3 promotes tumor development and progression. Mechanistically, the overexpression of *MST3* increases the phosphorylation of VAV2, and subsequently promotes VAV2-mediated activation of the Rac1-cyclin D1 signaling pathway that is required for the growth of breast cancer cells. Further investigation demonstrated that the proline-rich region of MST3 (K^353^DIPKRP^359^) interacts with the SH3 domain of VAV2, which is required for MST3-mediated promotion of proliferation of these cancer cells [4]. Lee et al. reported that the inhibition of *MST3* expression led to enhanced expression of cyclin-dependent kinase inhibitor p21, resulting in p21-mediated inhibition of cell cycle in human gastric cancer cell line MKN45, but not NCIN87 [7], suggesting that MST3 promotes tumor cell growth in a cell type-dependent manner. In addition, studies have reported an indirect role of MST3 in regulating the development of cancer. Nuclear Dbf2-related (NDR), a serine/threonine protein kinase, which directly phosphorylates p21 at S146, increases the progression of G1 by stabilizing c-Myc and preventing the accumulation of p21. Because NDR is the first identified substrate for MST3 (phosphorylates NDR at Thr444/Thr442) [51], it suggests that MST3 plays the oncogenic role by activating NDR. Furthermore, MST3 interacts with the evolutionarily conserved MO25 scaffolding protein [52], which is the master regulator of the LKB1 (serine-threonine liver kinase B1) tumor suppressor [53], indicating the possibility of MST3 in regulating tumor progression by targeting the MO25-LKB1 pathway. In view of the role of MST3 in promoting cancer development, Olesen et al. have discovered fourteen chemical compounds as MST3 inhibitors by using the kinase domain of MST3 (residues 1–303) to screen against the kinase inhibitor library from Selleck Chemicals (Table 4) [54]. This finding indicates that targeting MST3 with small-molecule inhibitors may be beneficial for controlling disease progression.

**Table 4:** Compounds confirmed as the MST3 inhibitors

Category	MST3 inhibitor	IC_50_ (μM)	Reference
Triazole diamines	CDK2 inhibitor III	0.014	[54]
	JNJ-7706621	1.3	
Triazole amine	Dasatinib	7.4	
Triazole phenol	TP fragment	0.023	
Quinoline and quinazoline amines	Bosutinib	0.003	
	Saracatinib	11	
Pyrazoles	Danusertib	0.16	
	AT9283	0.46	
Indolinones	Hesperadin	0.01	
	C16	0.019	
	Sunitinib	0.21	
Aminopyrimidine	PF-03814735	0.023	
Pyrazolopyrididine	PP-121	0.086	
Benzimidazole	CP-673451	0.26	

Although MST3 has been previously recognized as a pro-tumoral protein, the function of MST3 to inhibit tumor progression has been reported. Luo et al. reported that the down-expression of *MST3* by the oncogene MiR-222 that directly binds to the promoter region of *MST3* promotes the migration and invasion of colorectal cancer cell line [55]. Mechanistically, the suppression of endogenous MST3 enhances the cellular migration in human adenocarcinoma cells, MCF-7, by increasing the phosphorylation of paxillin by a protein tyrosine phosphatase PTP-PEST [28]. The suppression of *STK24* increased cell migration by inhibiting CDH1 (E-Cadherin) and enhancing the levels of CD44 in gastric cancer cells [38]. In addition, an in vivo study reported that the suppression of CD4^+^ T cells increased tumor metastasis and growth in an *STK24*-silenced mouse model of gastric cancer while enhancing the expansion of CD11b^+^Ly6C^+^ MDSCs and F4/80^+^ TAMs [38, 39].

### MST3 and CNS development

Proper neuronal migration during cortical development is required for normal neuronal function. It is reported that MST4 (STK25), a GCKIII family member, promotes neuronal migration in the neocortex by balancing the Rac1 activity and RhoA levels through the formation of complexes with α-PIX and β-PIX, GTPase regulatory enzymes, and Cullin3-Bacurd1/Kctd13 [8]. Although the conditional knockout of the *STK25* gene during mouse embryogenesis causes anomalous neuronal migration in the neocortex, it did not cause a cortical phenotype, indicating the existence of a complementary mechanism [8]. In their subsequent study, they found that MST3 compensates MST4 to regulate neuronal migration and polarization by modulating the activity of Rho GTPases [8]. Another study reported that MST3 is highly expressed in the developing mouse brain. The overexpression of *MST3* contributed to radial neuronal migration and final neuronal positioning in the developing mouse neocortex. Mechanistically, MST3 regulates neuronal migration by negatively regulating Rho-GTPase activity of RhoA, because RhoA plays a critical role in actin cytoskeletal reorganization by phosphorylating RhoA at Ser26 [31]. The phosphorylation of MST3 by Cdk5 at Ser79 is essential for its kinase activity and function in neuronal morphogenesis and migration [31]. Moreover, MST3 is necessary for the proper development of filopodia, dendritic spine, and excitatory synapse in the CNS. The MST3-mediated promotion of dendritic filopodia and spine synapse development occurs through the phosphorylation of TAO1/2 kinases [44].

Recently, a neuron-specific homolog of MST3 (isoform a), termed MST3b (isoform b), was reported. Amino acid sequence alignment revealed that the six N-terminal amino acids of MST3b are different from that of the canonical MST3. The further functional investigation demonstrated that the MST3b is essential for the development and repair of brain circuitry by promoting axon outgrowth [56]. Injured spinal cord neurons have increased levels of MST3b which promotes neuronal regeneration through the activation of P42/44MAPK and LIMK/Cofilin signaling pathways [45].

## Conclusion

MST3 has emerged as a pleiotropic regulator in modulating a variety of biological functions, such as apoptosis, immune signaling, metabolism, hypertension, tumor progression, and CNS development. The function of MST3-mediated regulation is closely related to protein activity-associated activities, such as protein cleavage, post-transcriptional modification, subcellular distribution, and interactions with its several adaptor proteins. Therefore, targeting MST3 and its activity-associated characteristics can be used as a potential therapeutic strategy for controlling the progression of various disease processes.

## Data Availability

The data will be provided on contacting corresponding author.
